# Supplementary figures and images for: Architecture of genome-wide transcriptional regulatory network reveals dynamic functions and evolutionary trajectories in Pseudomonas syringae
Source: eLife. 2025 Mar 31;13:RP96172. doi: 10.7554/eLife.96172 (PMC11957545; doi:10.7554/eLife.96172)

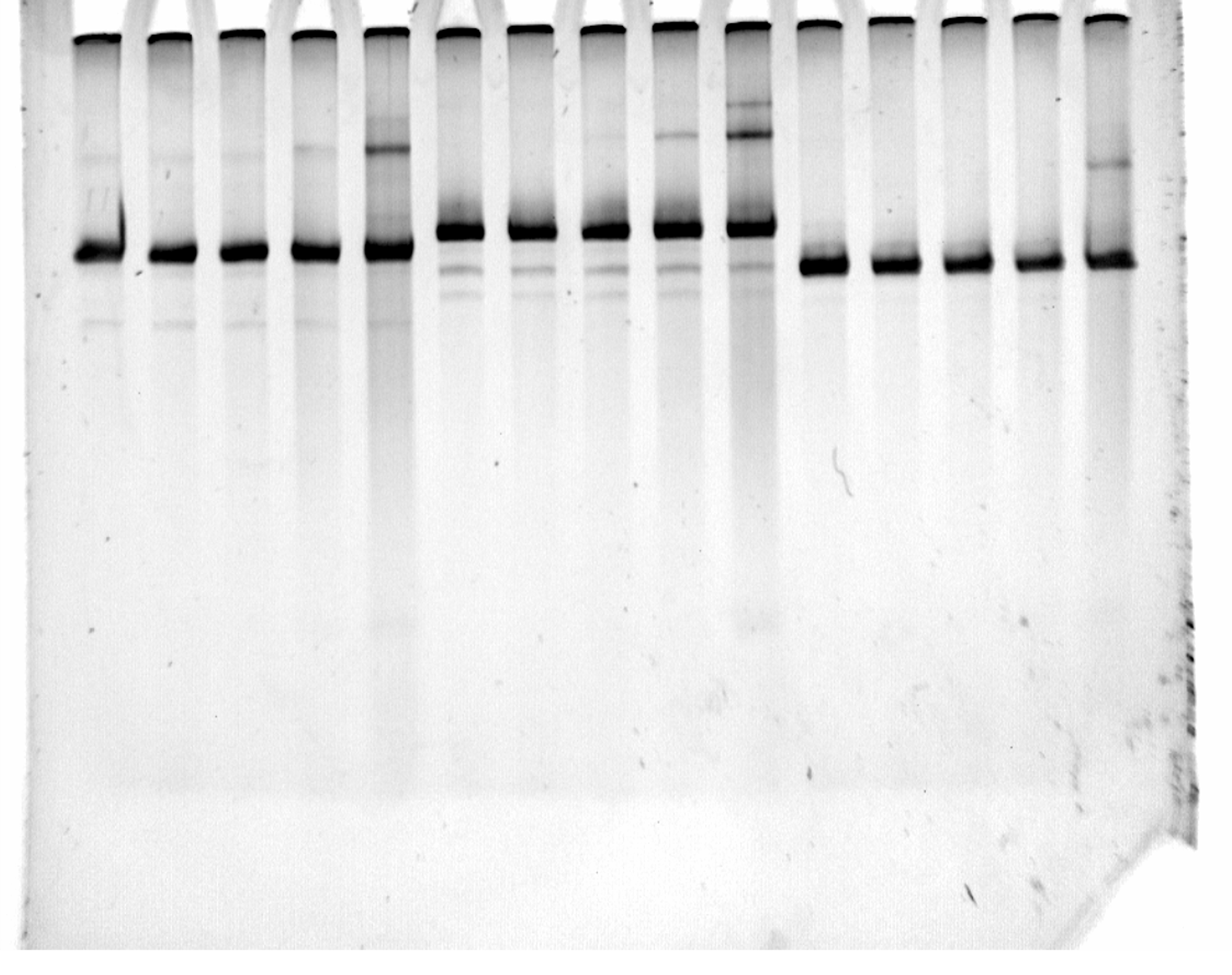

Supplement: Figure 3—source data 2. [file elife-96172-fig3-data2.zip › Figure 3-source data 2/1951-hrpR-hopAE1-hopAH2.tif]

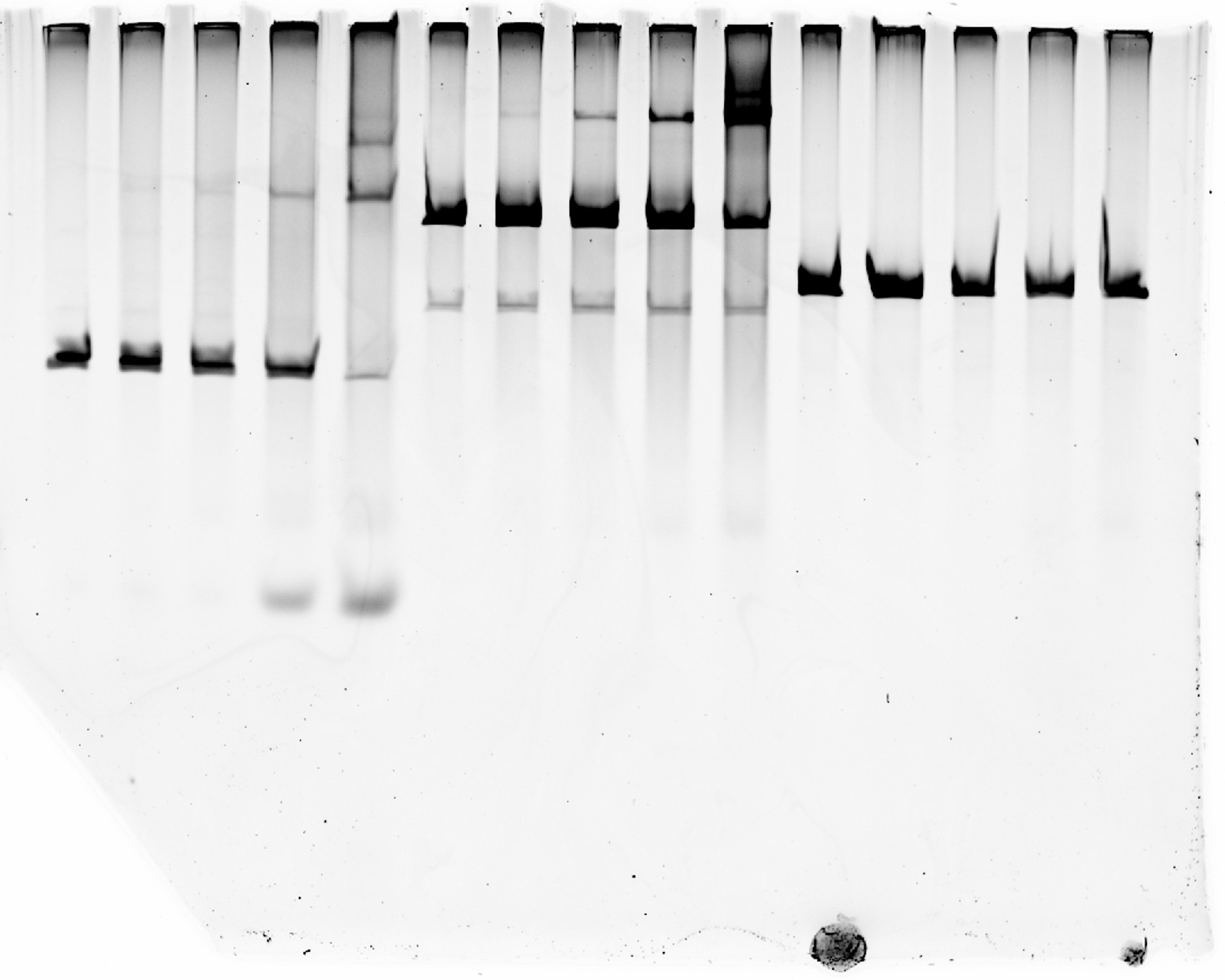

Supplement: Figure 3—source data 2. [file elife-96172-fig3-data2.zip › Figure 3-source data 2/2193-fleQ.tif]

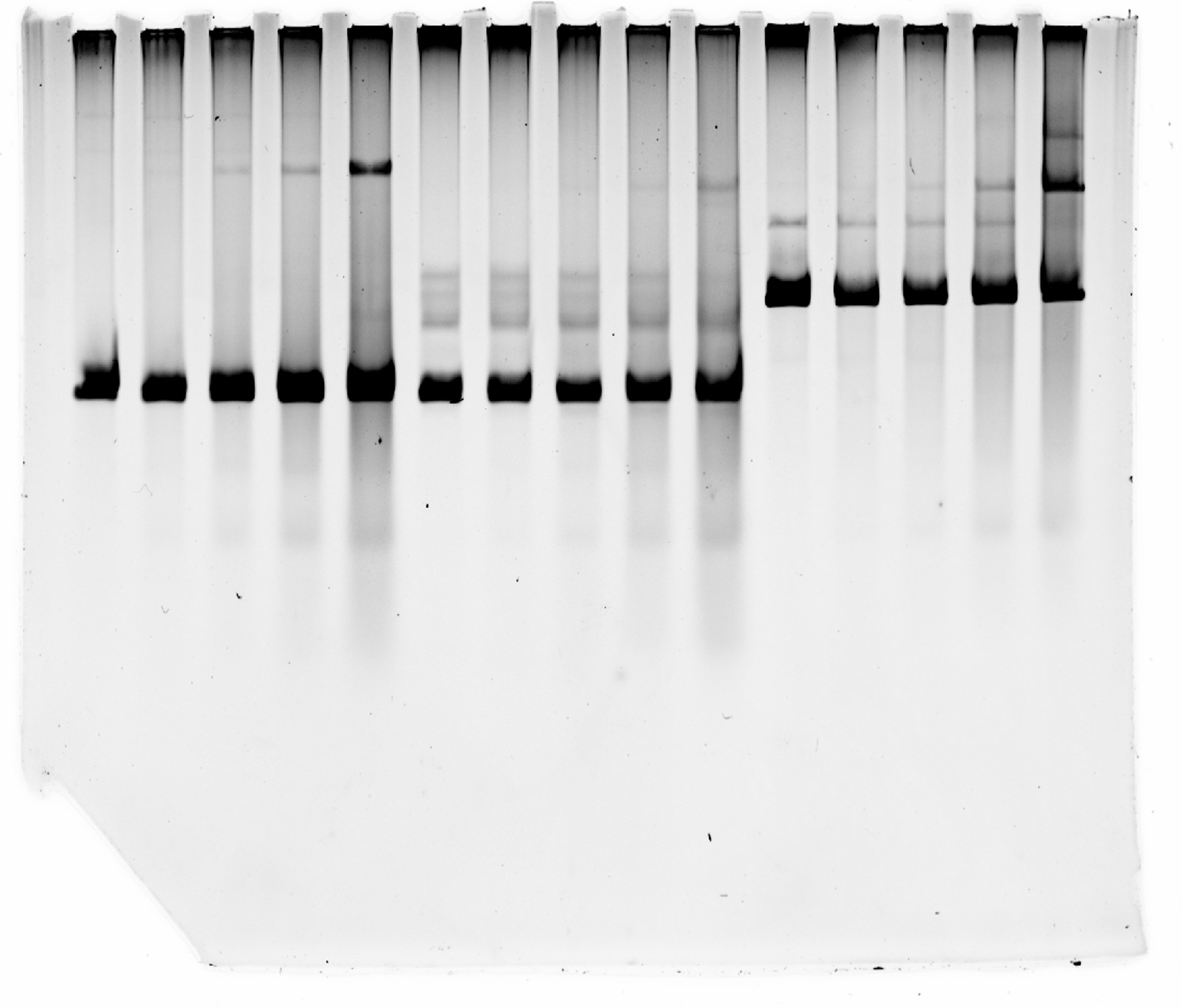

Supplement: Figure 3—source data 2. [file elife-96172-fig3-data2.zip › Figure 3-source data 2/2193-flhF.tif]

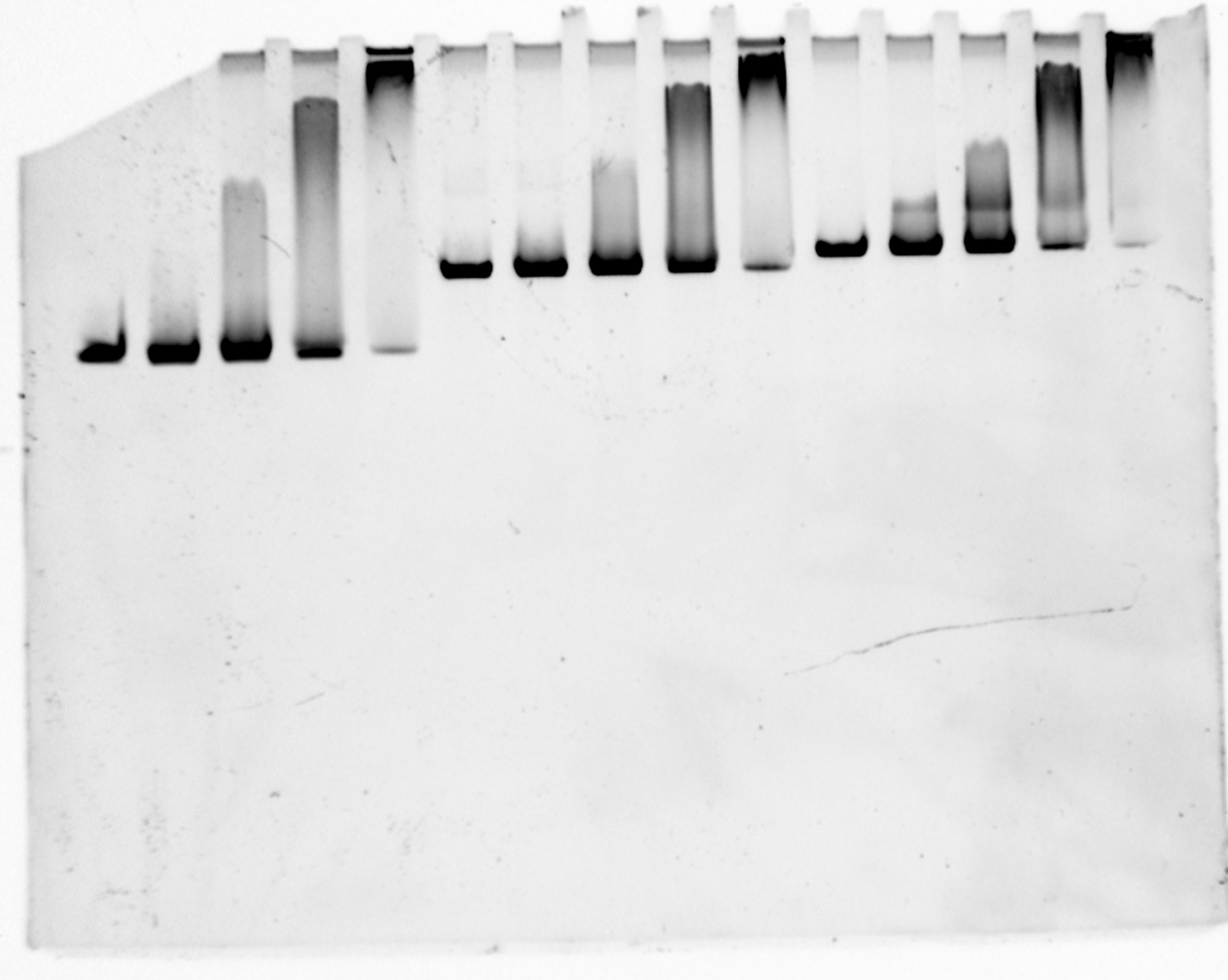

Supplement: Figure 3—source data 2. [file elife-96172-fig3-data2.zip › Figure 3-source data 2/3268-alg44.tif]

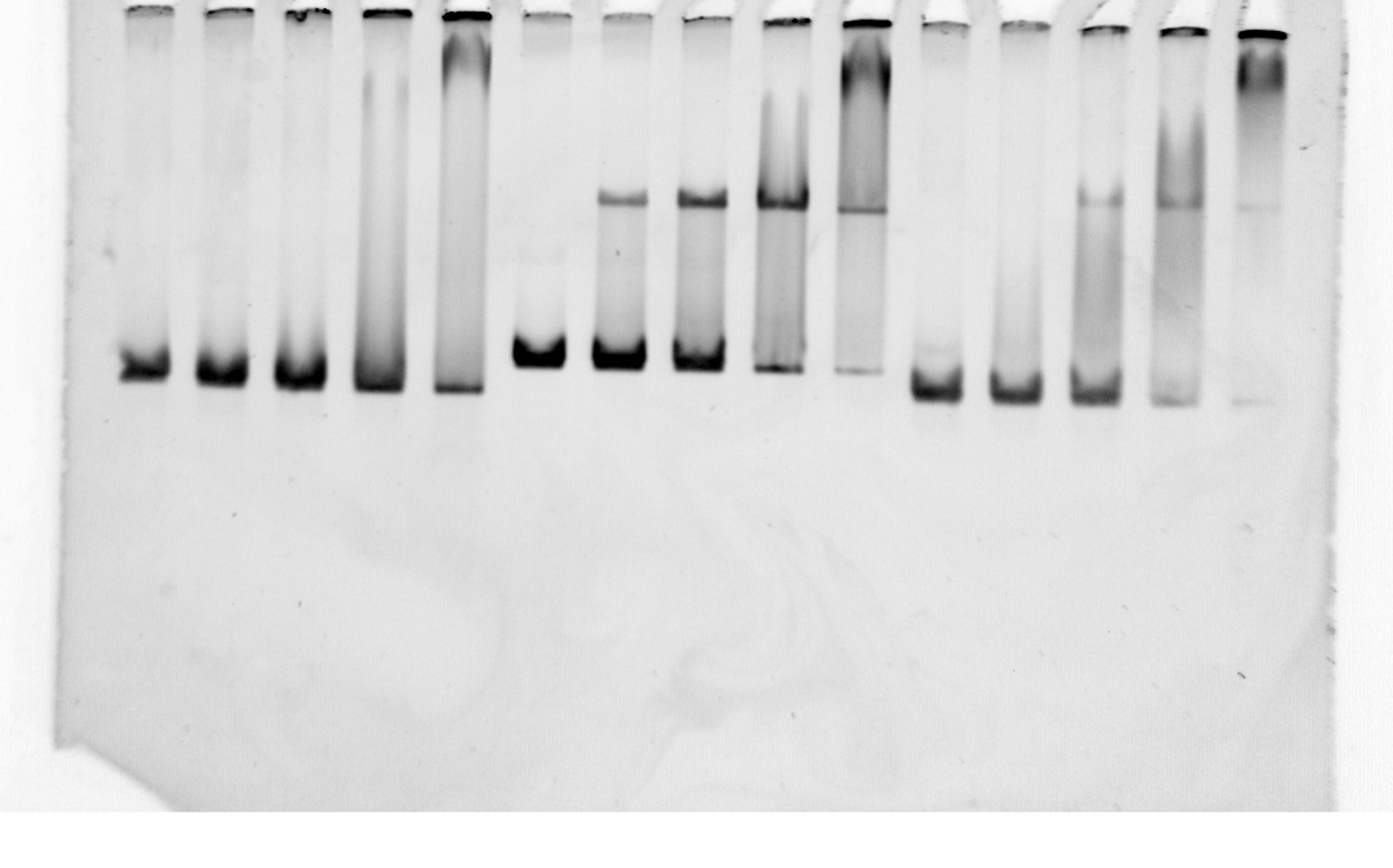

Supplement: Figure 3—source data 2. [file elife-96172-fig3-data2.zip › Figure 3-source data 2/3268-hrpR.tif]

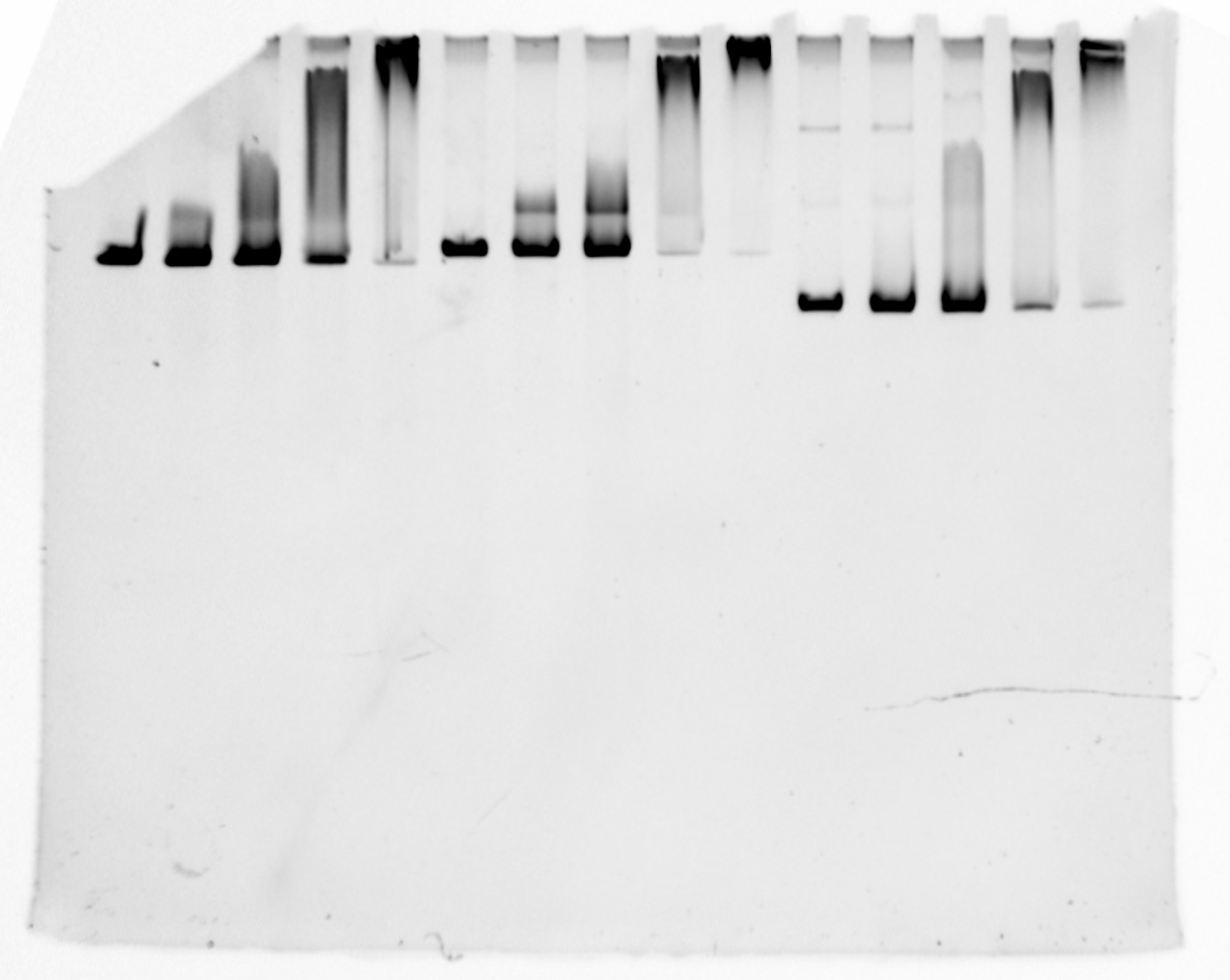

Supplement: Figure 3—source data 2. [file elife-96172-fig3-data2.zip › Figure 3-source data 2/3268-pilM.tif]

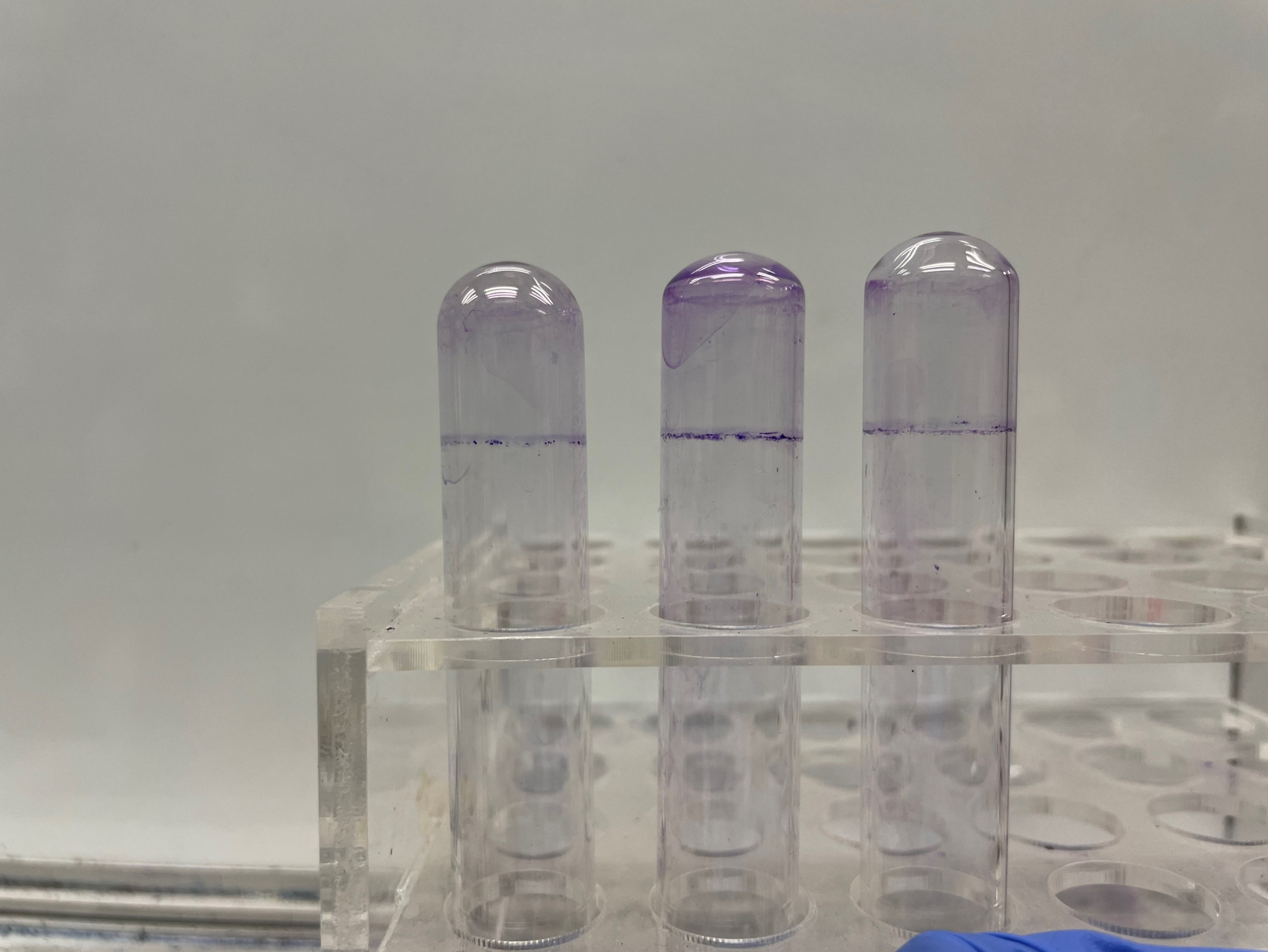

Supplement: Figure 3—source data 2. [file elife-96172-fig3-data2.zip › Figure 3-source data 2/biofilm.tif]

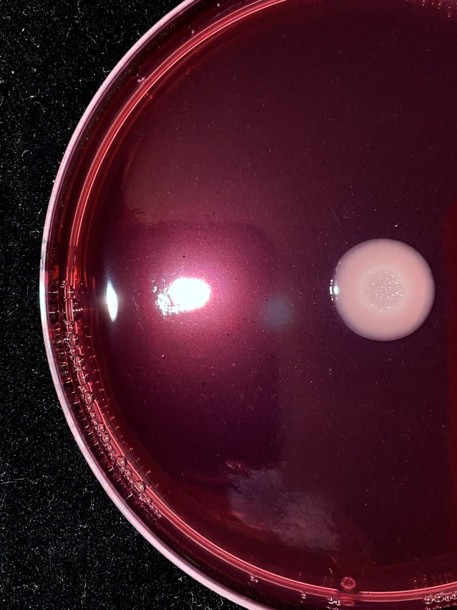

Supplement: Figure 3—source data 2. [file elife-96172-fig3-data2.zip › Figure 3-source data 2/EPS-3268 complementary strain.tif]

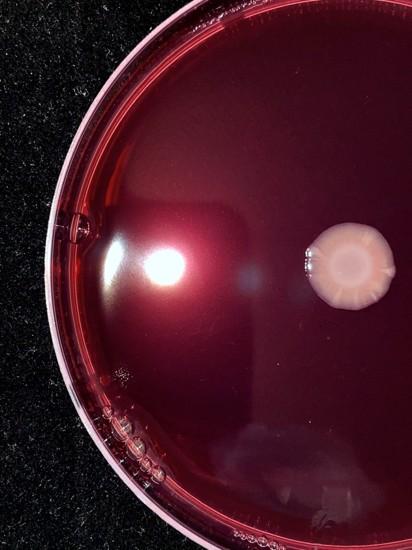

Supplement: Figure 3—source data 2. [file elife-96172-fig3-data2.zip › Figure 3-source data 2/EPS-3268 mutant.tif]

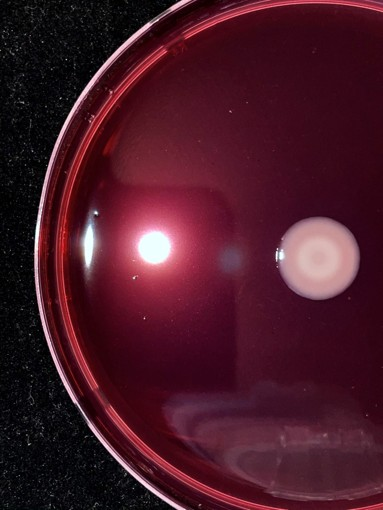

Supplement: Figure 3—source data 2. [file elife-96172-fig3-data2.zip › Figure 3-source data 2/EPS-WT.tif]

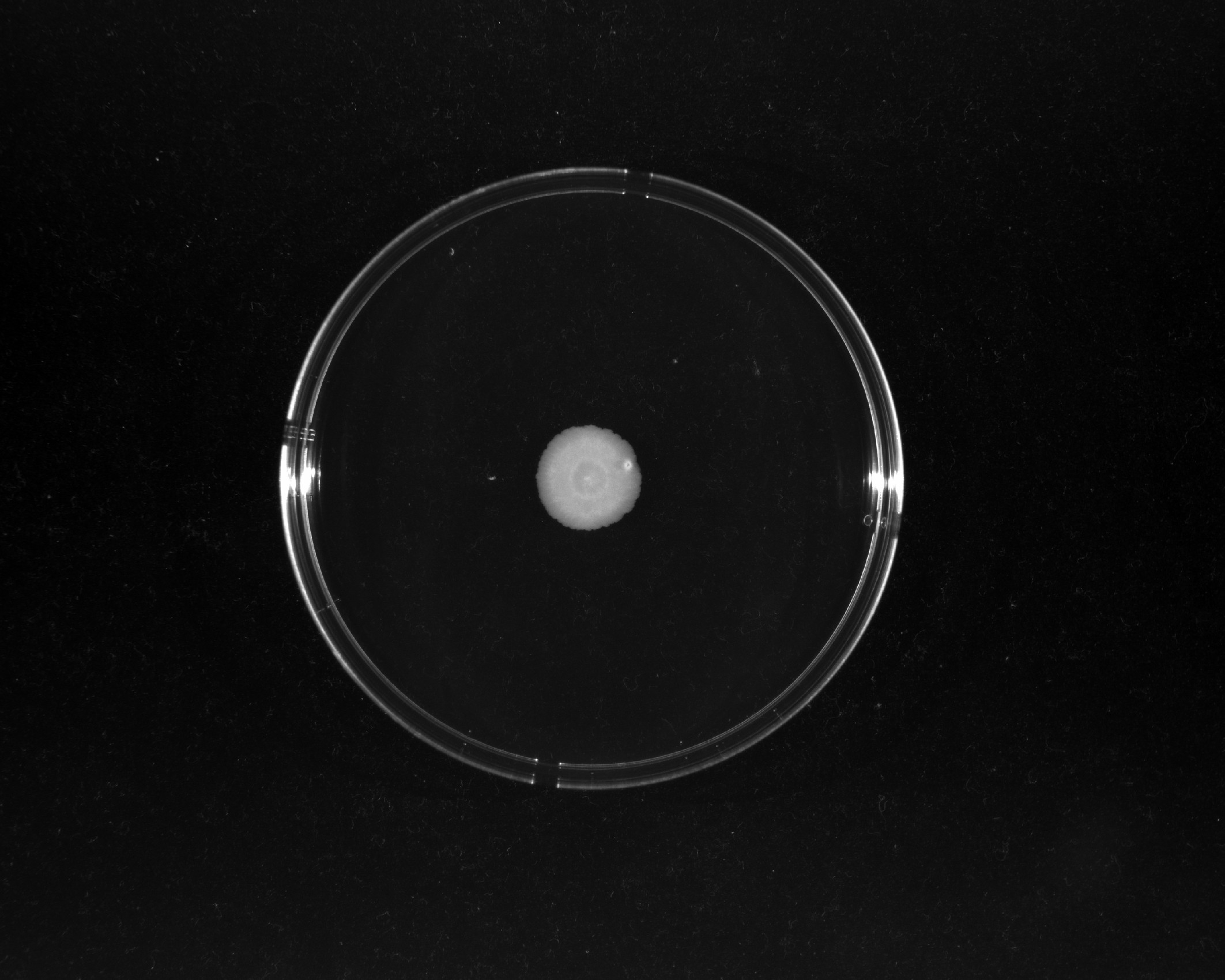

Supplement: Figure 3—source data 2. [file elife-96172-fig3-data2.zip › Figure 3-source data 2/motility-2193 complementary strain.tif]

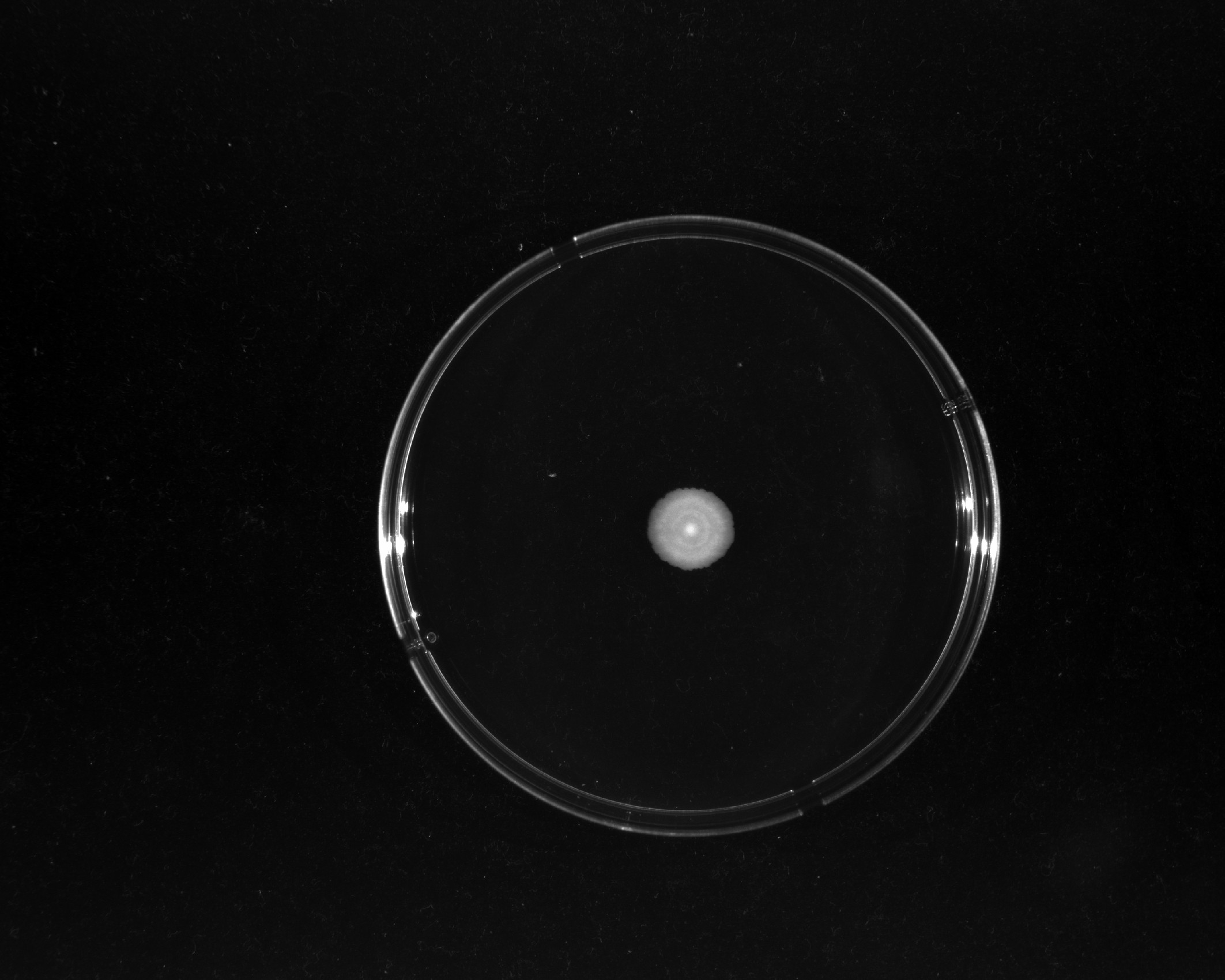

Supplement: Figure 3—source data 2. [file elife-96172-fig3-data2.zip › Figure 3-source data 2/motility-2193 mutant.tif]

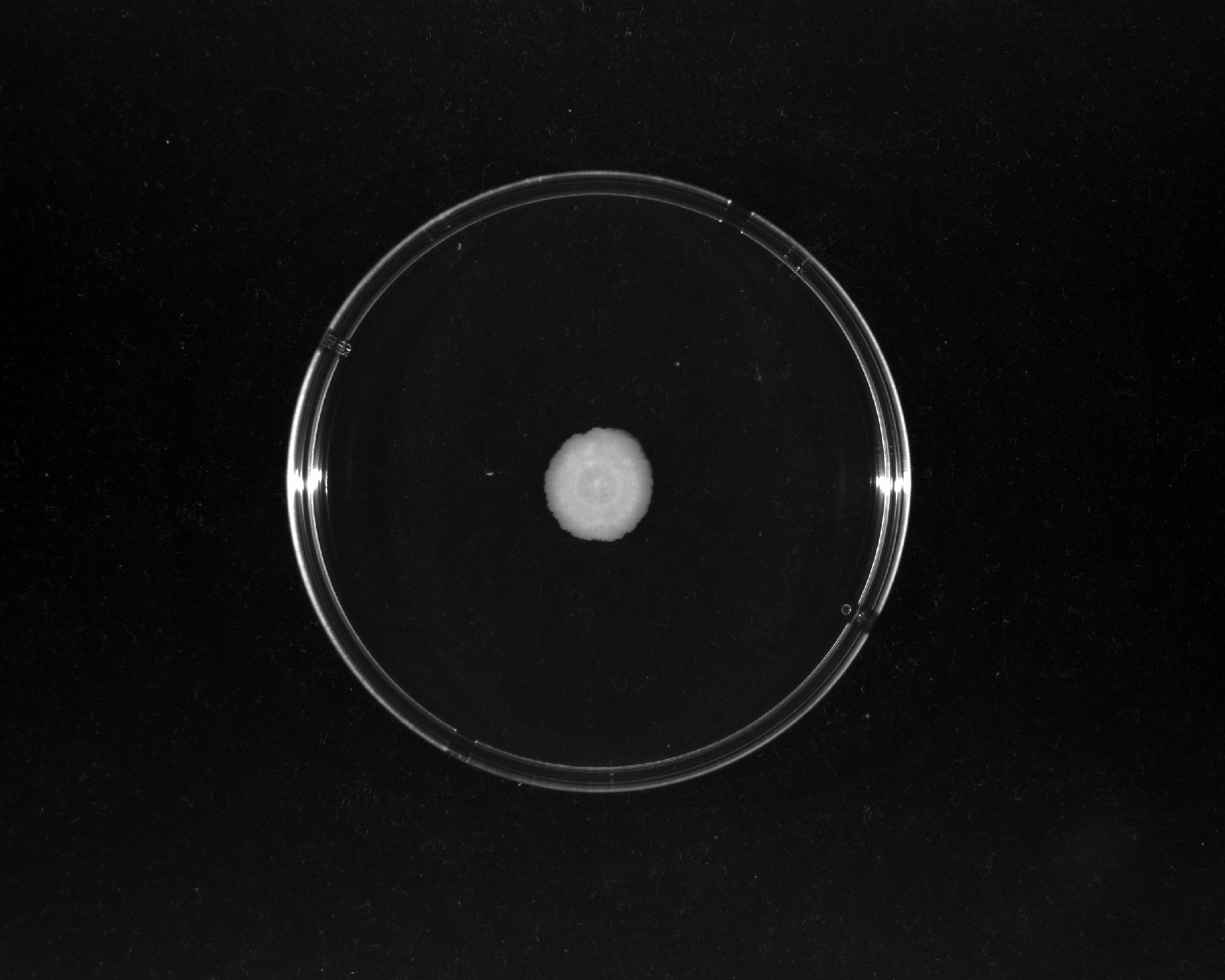

Supplement: Figure 3—source data 2. [file elife-96172-fig3-data2.zip › Figure 3-source data 2/motility-WT.tif]

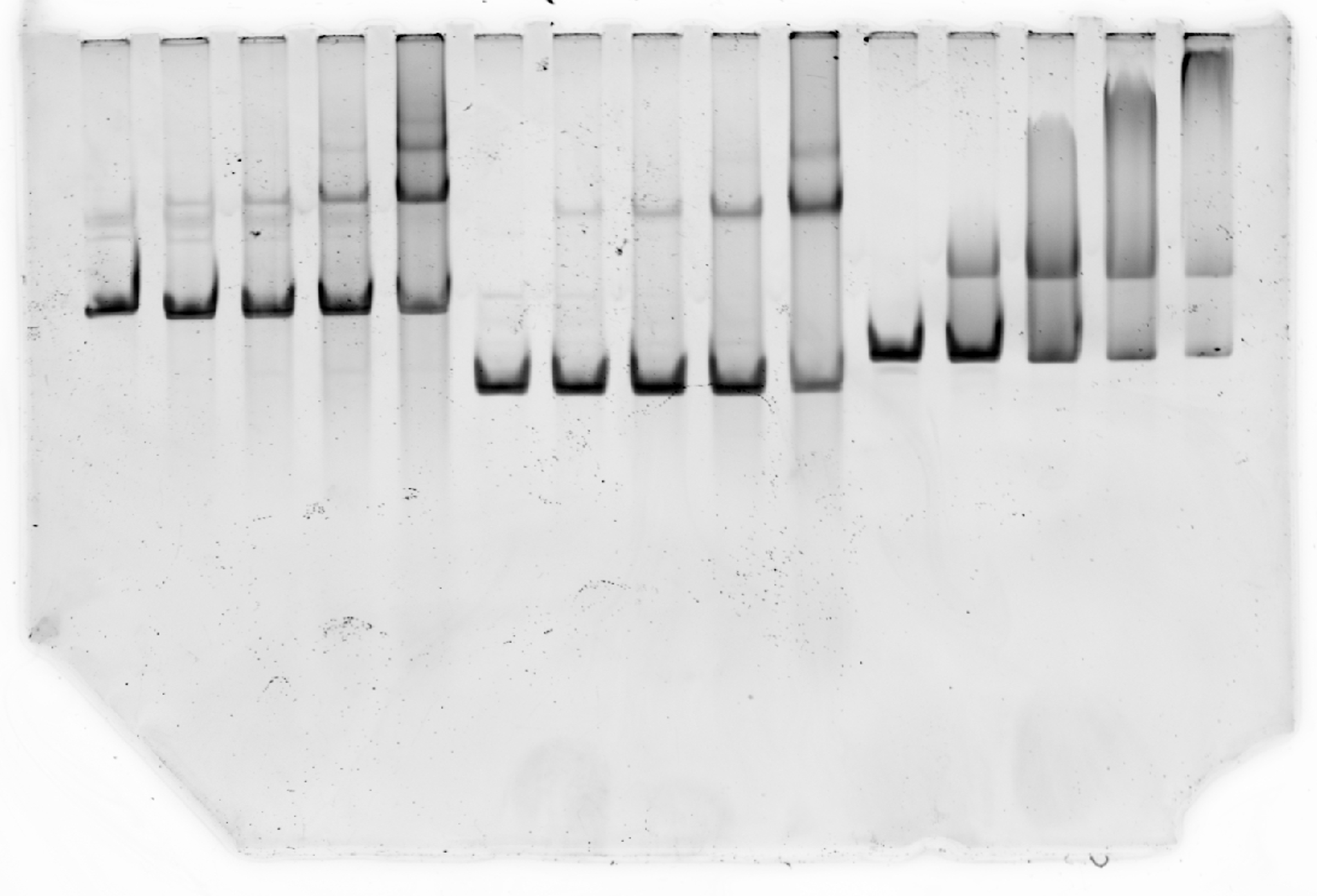

Supplement: Figure 3—figure supplement 1—source data 2. [file elife-96172-fig3-figsupp1-data2.zip › Figure 3-figure supplement 1-source data 2/1951-pilF.tif]

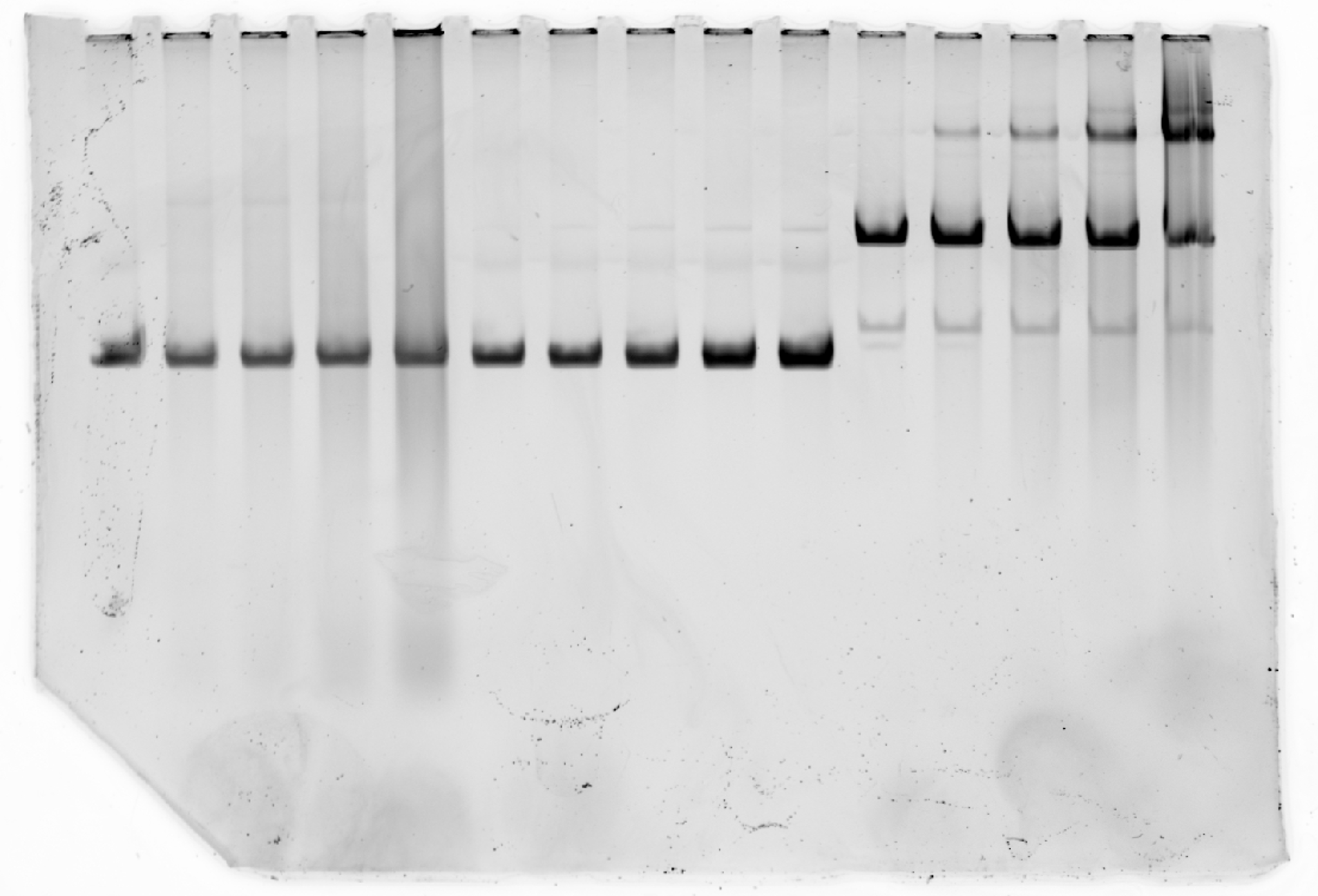

Supplement: Figure 3—figure supplement 1—source data 2. [file elife-96172-fig3-figsupp1-data2.zip › Figure 3-figure supplement 1-source data 2/1951-pilG.tif]

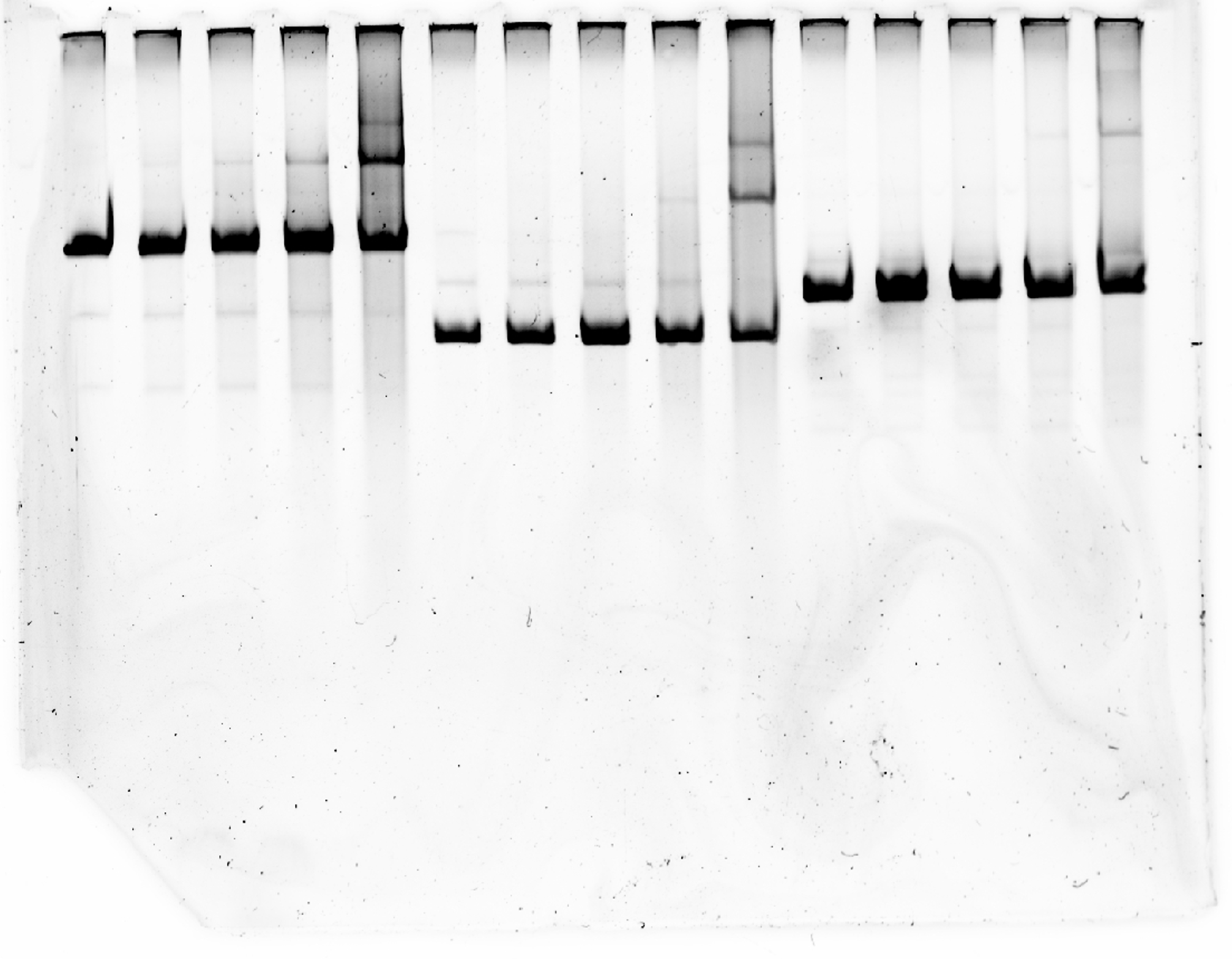

Supplement: Figure 3—figure supplement 1—source data 2. [file elife-96172-fig3-figsupp1-data2.zip › Figure 3-figure supplement 1-source data 2/1951-pilZ.tif]

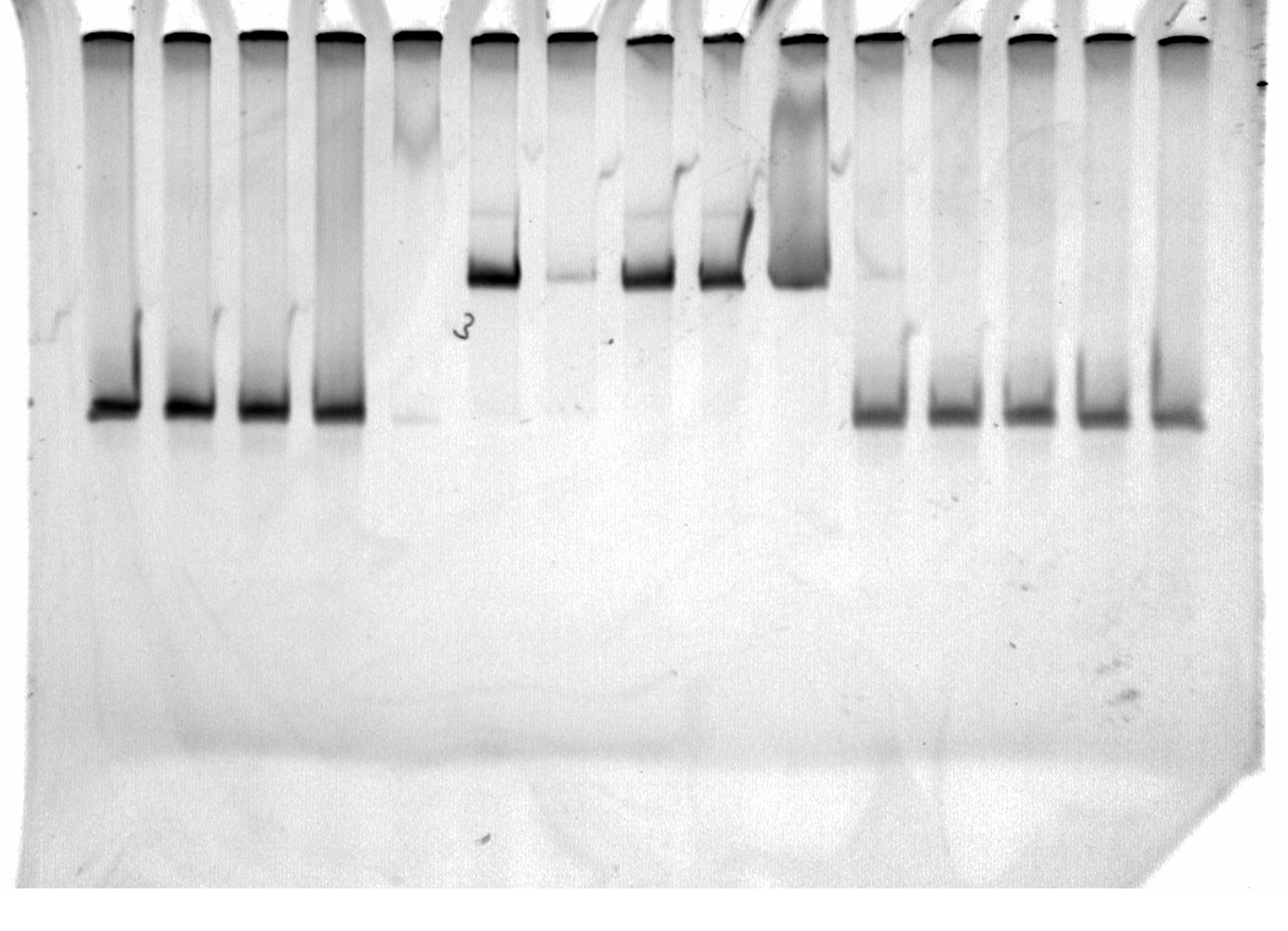

Supplement: Figure 3—figure supplement 1—source data 2. [file elife-96172-fig3-figsupp1-data2.zip › Figure 3-figure supplement 1-source data 2/1951-rpoD.tif]

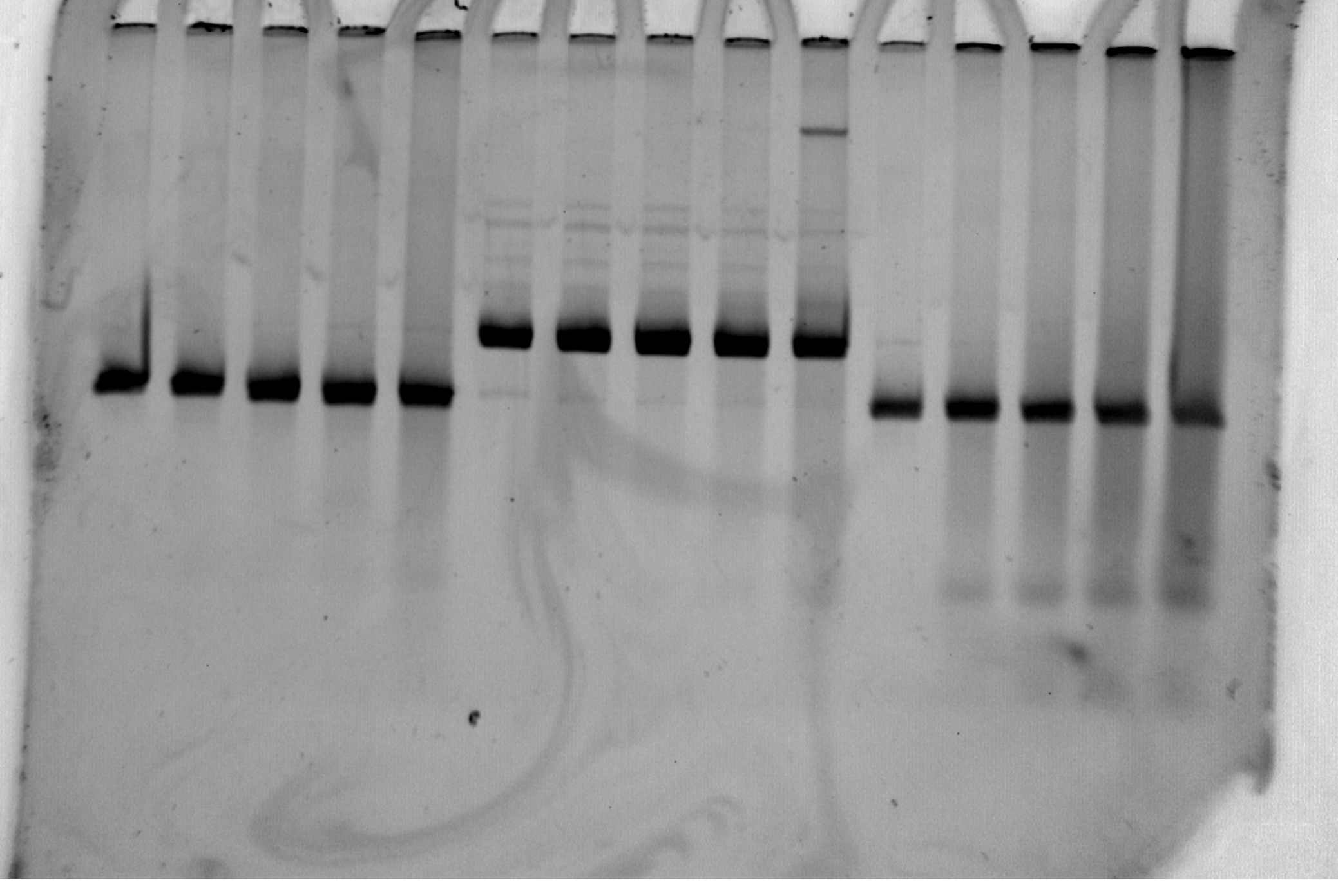

Supplement: Figure 3—figure supplement 1—source data 2. [file elife-96172-fig3-figsupp1-data2.zip › Figure 3-figure supplement 1-source data 2/2193-rpoD.tif]

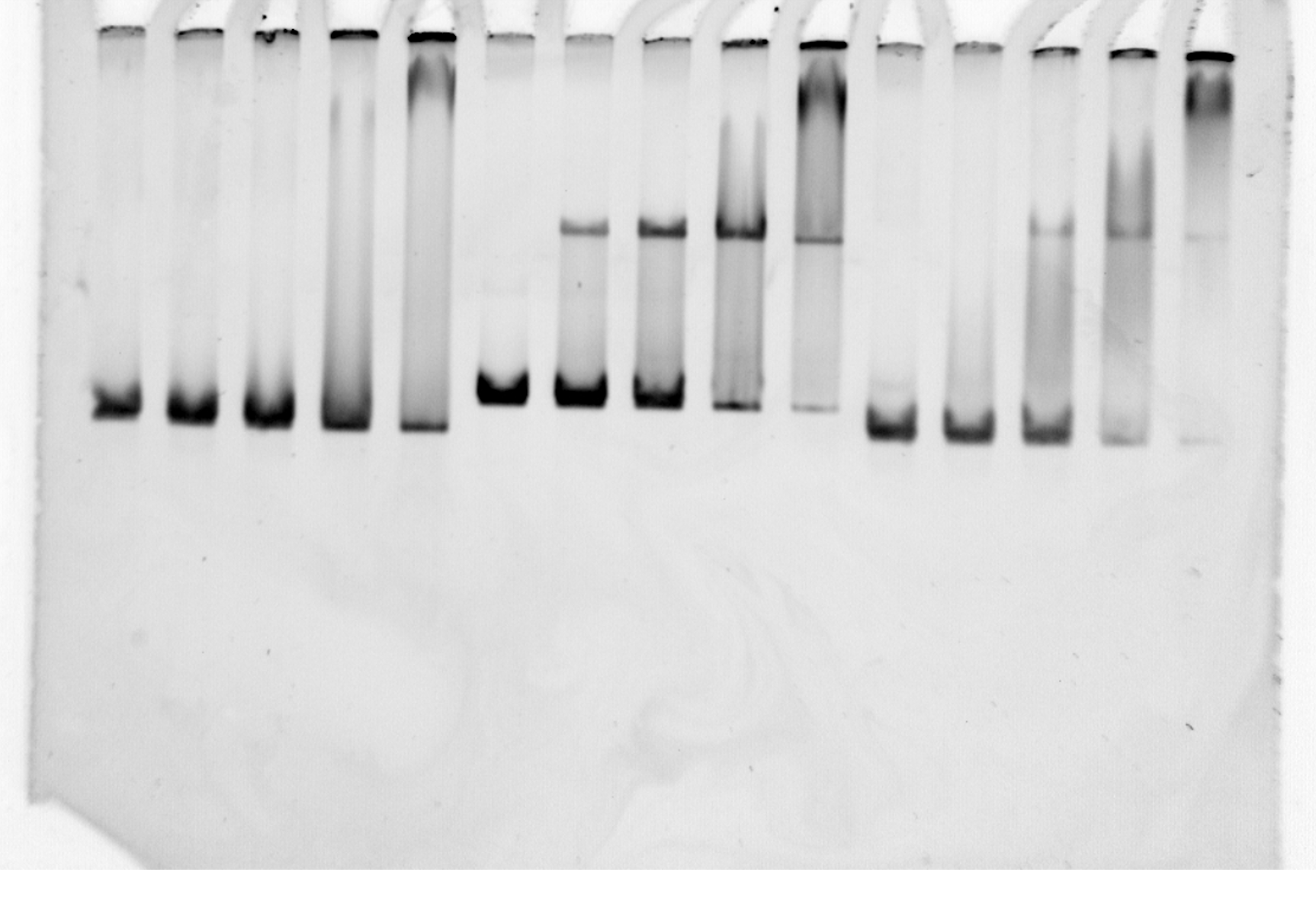

Supplement: Figure 3—figure supplement 1—source data 2. [file elife-96172-fig3-figsupp1-data2.zip › Figure 3-figure supplement 1-source data 2/3268-3658.tif]

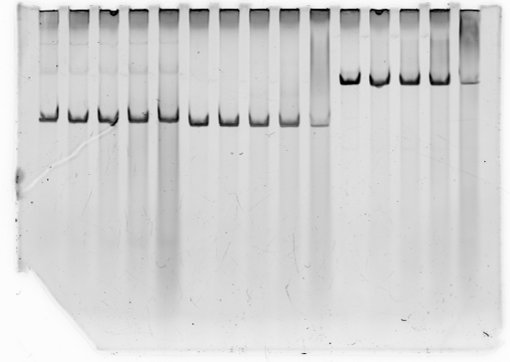

Supplement: Figure 3—figure supplement 1—source data 2. [file elife-96172-fig3-figsupp1-data2.zip › Figure 3-figure supplement 1-source data 2/3798-4518.tif]

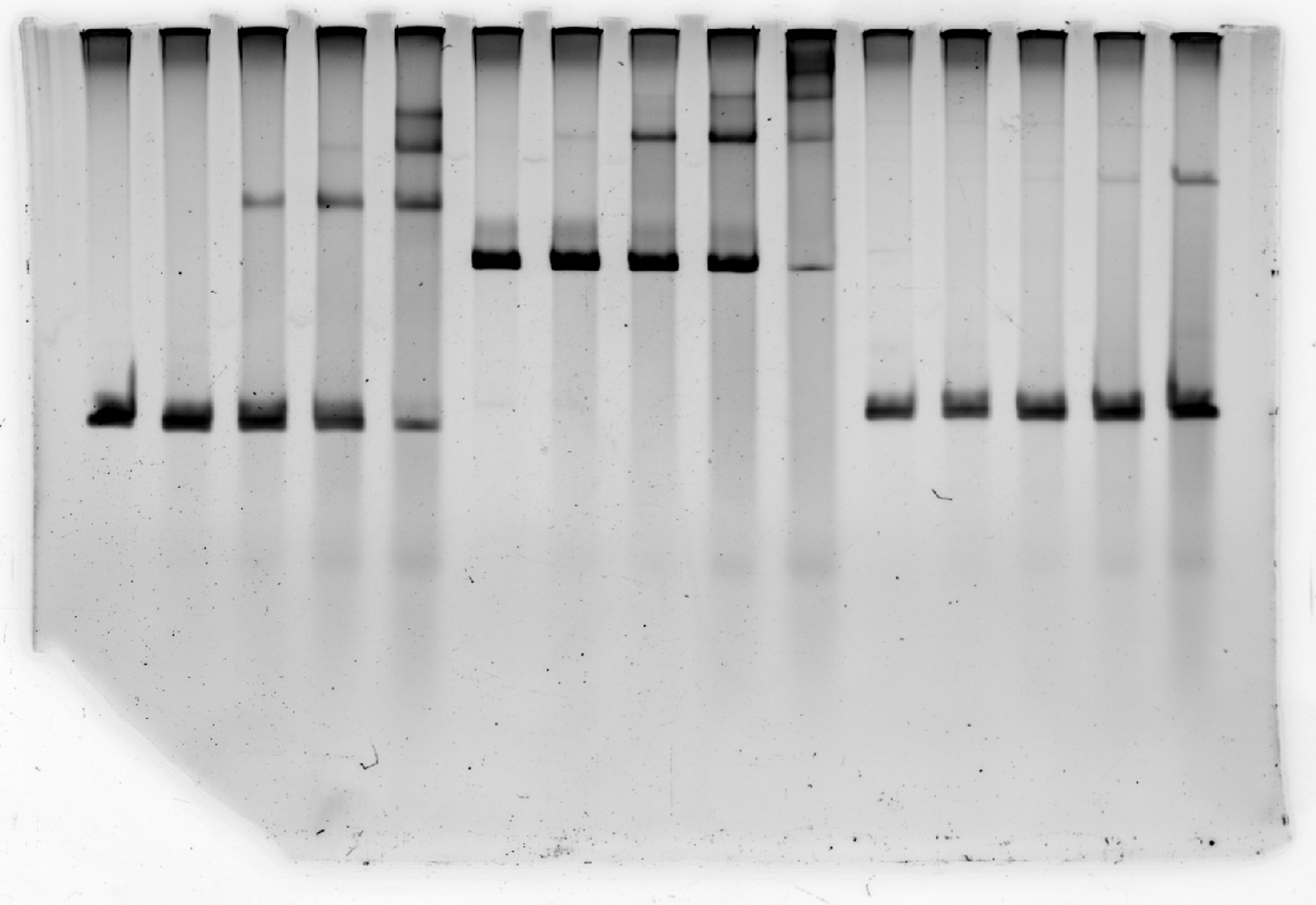

Supplement: Figure 3—figure supplement 1—source data 2. [file elife-96172-fig3-figsupp1-data2.zip › Figure 3-figure supplement 1-source data 2/3798-filD-fleQ.tif]

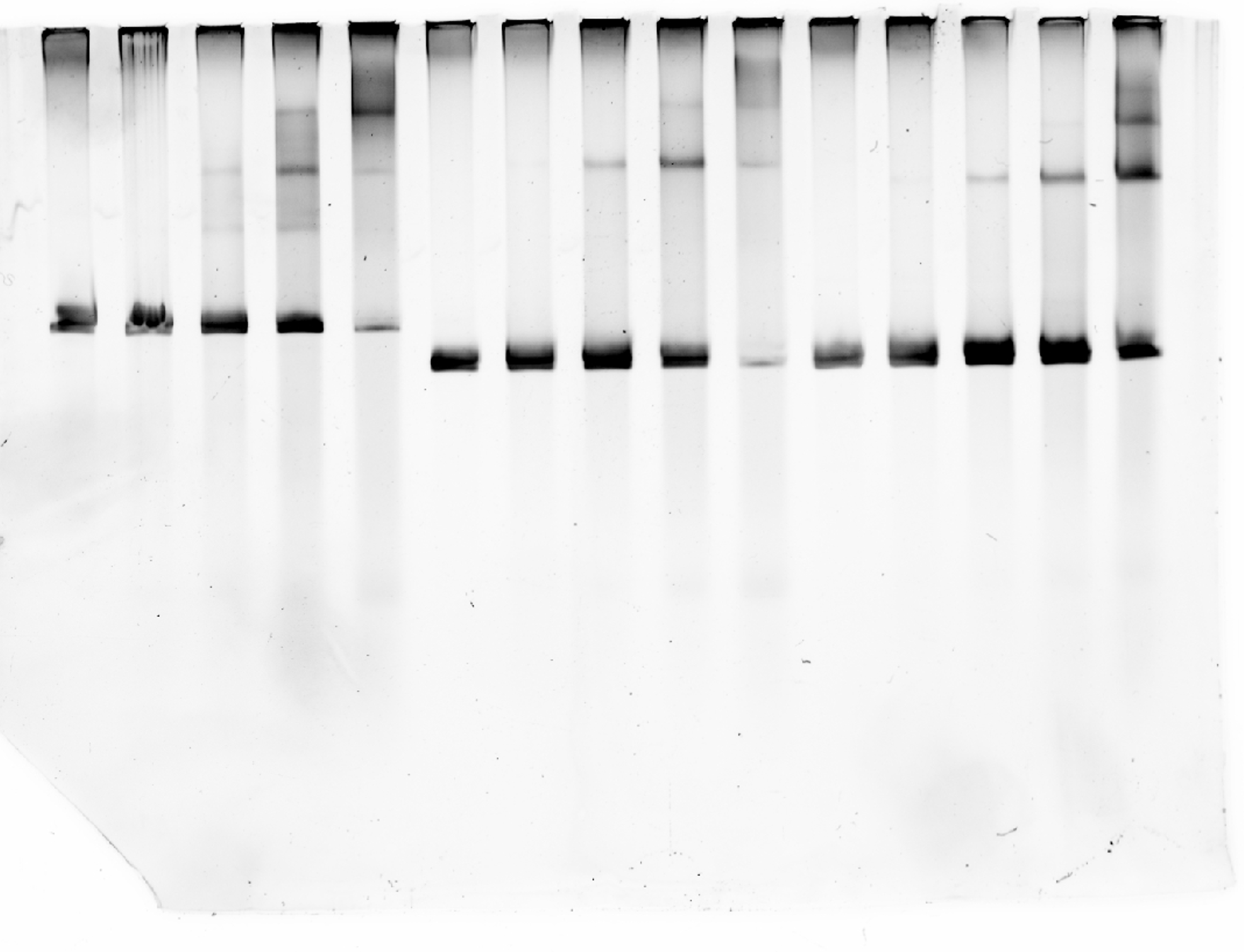

Supplement: Figure 3—figure supplement 1—source data 2. [file elife-96172-fig3-figsupp1-data2.zip › Figure 3-figure supplement 1-source data 2/3798-filK-fliE.tif]

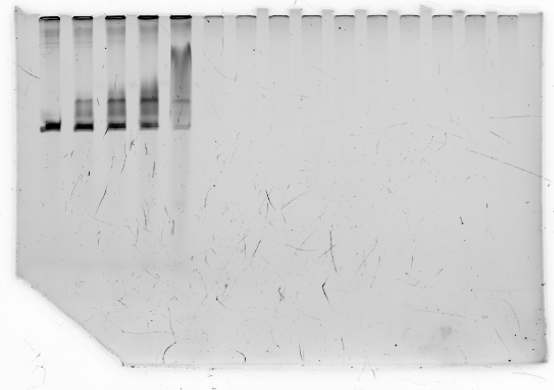

Supplement: Figure 4—figure supplement 2—source data 2. [file elife-96172-fig4-figsupp2-data2.zip › Figure 4-figure supplement 2-source data 2/0755-3109.tif]

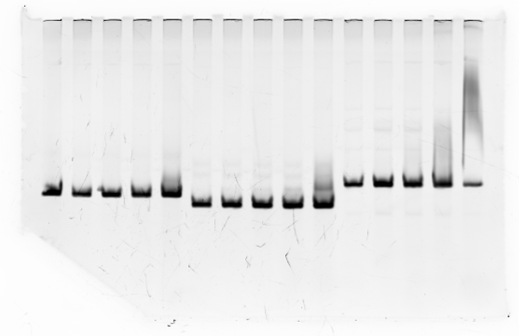

Supplement: Figure 4—figure supplement 2—source data 2. [file elife-96172-fig4-figsupp2-data2.zip › Figure 4-figure supplement 2-source data 2/0755-4598 5210.tif]

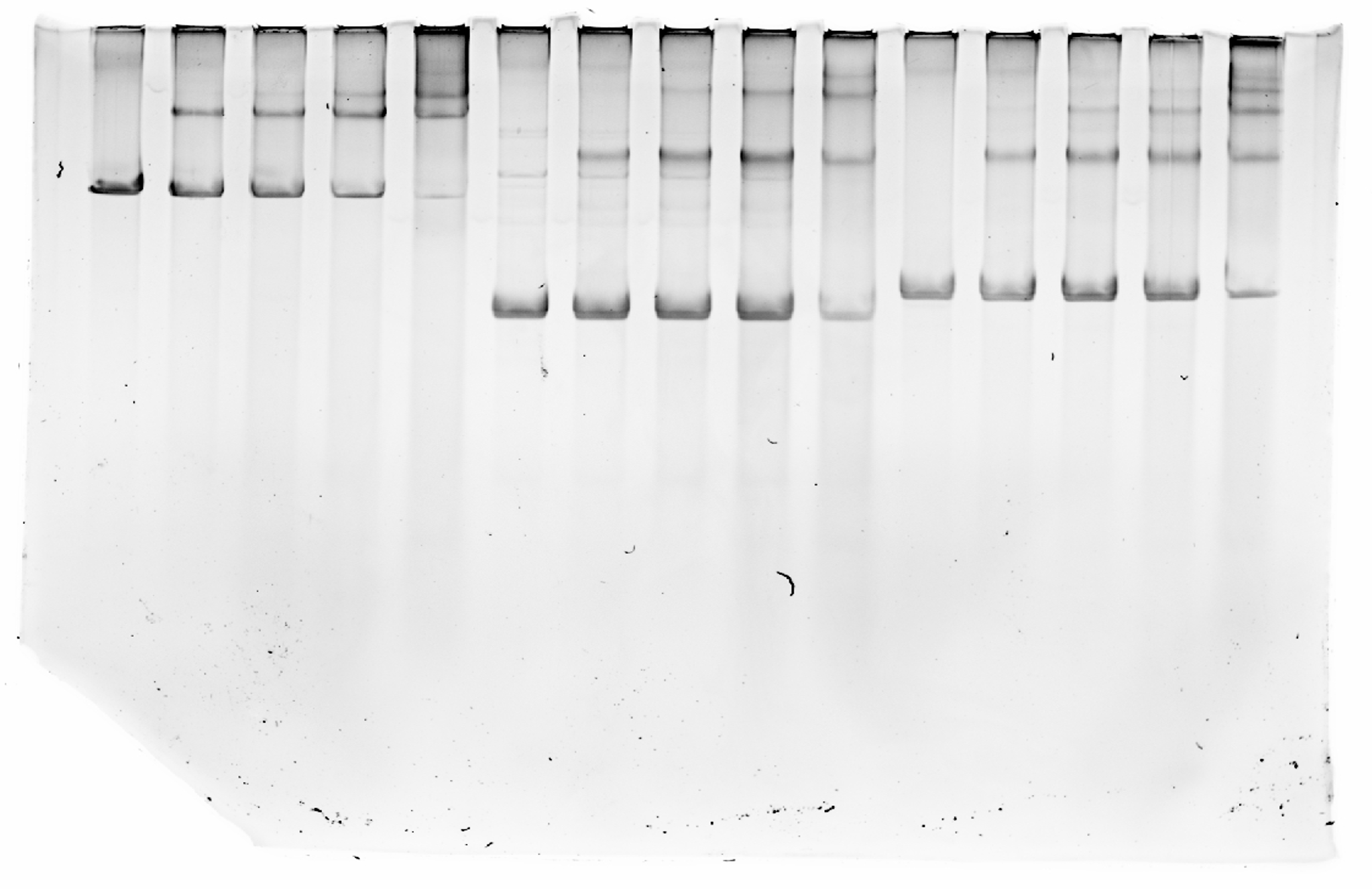

Supplement: Figure 4—figure supplement 2—source data 2. [file elife-96172-fig4-figsupp2-data2.zip › Figure 4-figure supplement 2-source data 2/3798-3881 5119.tif]

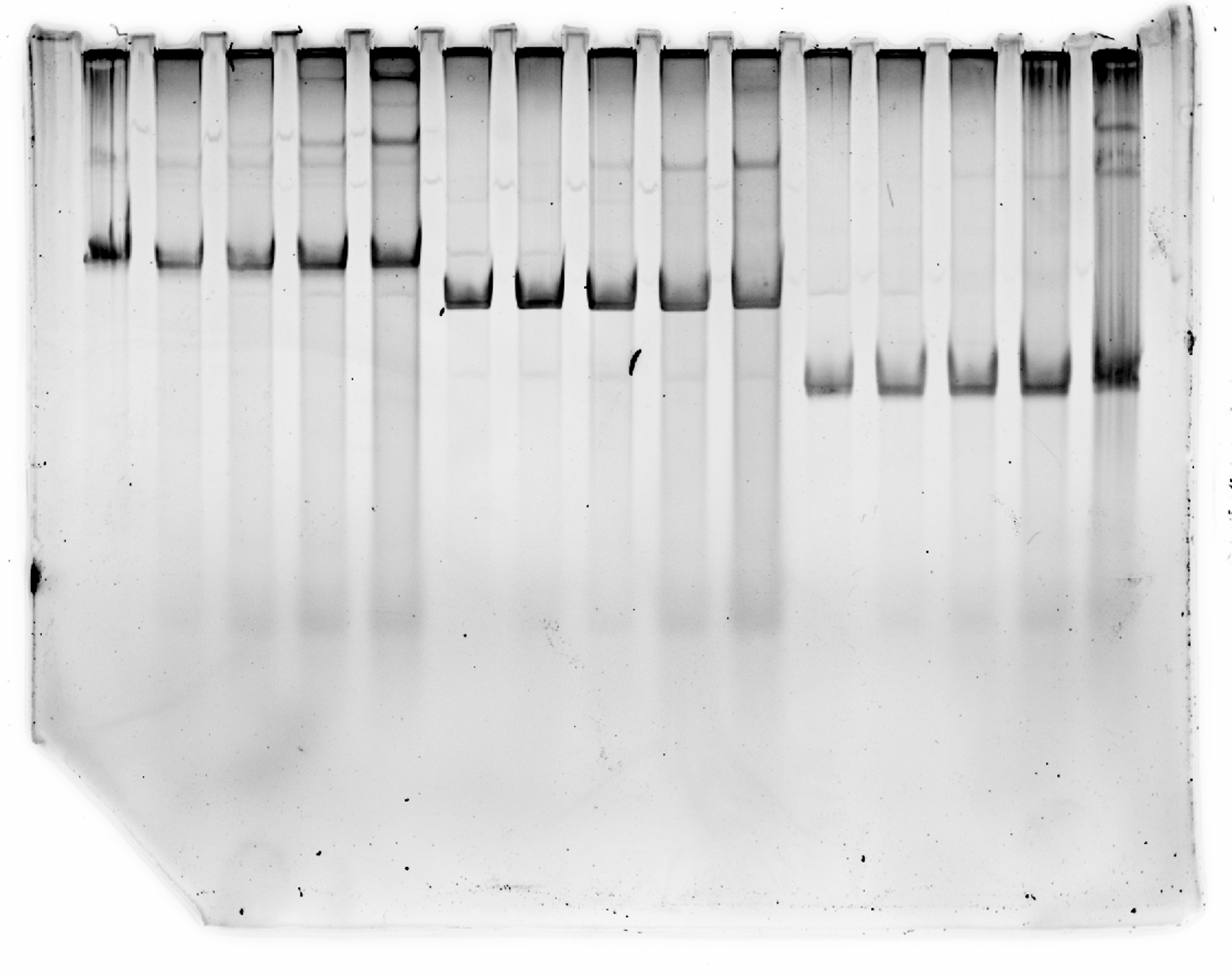

Supplement: Figure 4—figure supplement 2—source data 2. [file elife-96172-fig4-figsupp2-data2.zip › Figure 4-figure supplement 2-source data 2/4638-0550.tif]

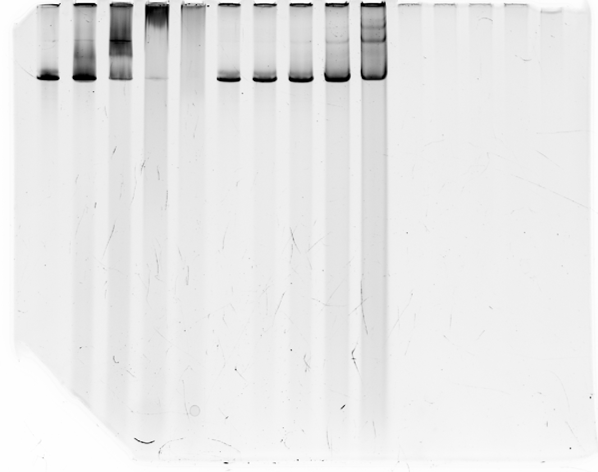

Supplement: Figure 4—figure supplement 2—source data 2. [file elife-96172-fig4-figsupp2-data2.zip › Figure 4-figure supplement 2-source data 2/4638-3881.tif]

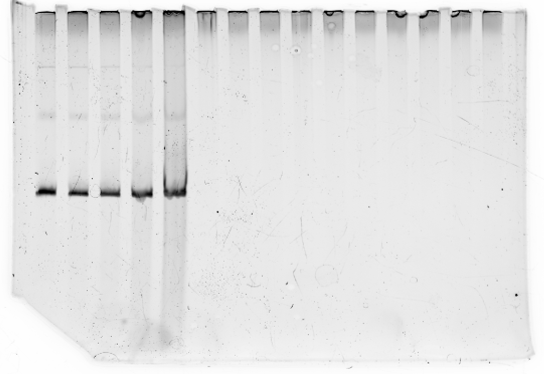

Supplement: Figure 4—figure supplement 2—source data 2. [file elife-96172-fig4-figsupp2-data2.zip › Figure 4-figure supplement 2-source data 2/4638-4117.tif]
