# Supplementary material for: Architecture of genome-wide transcriptional regulatory network reveals dynamic functions and evolutionary trajectories in Pseudomonas syringae
Source: eLife. 2025 Mar 31;13:RP96172. doi: 10.7554/eLife.96172 (PMC11957545; doi:10.7554/eLife.96172)
Supplement: Figure 4—figure supplement 2—source data 1. [file elife-96172-fig4-figsupp2-data1.zip › Figure 4-figure supplement 2-source data 1/Figure 4-figure supplement 2-source data 1.pdf]

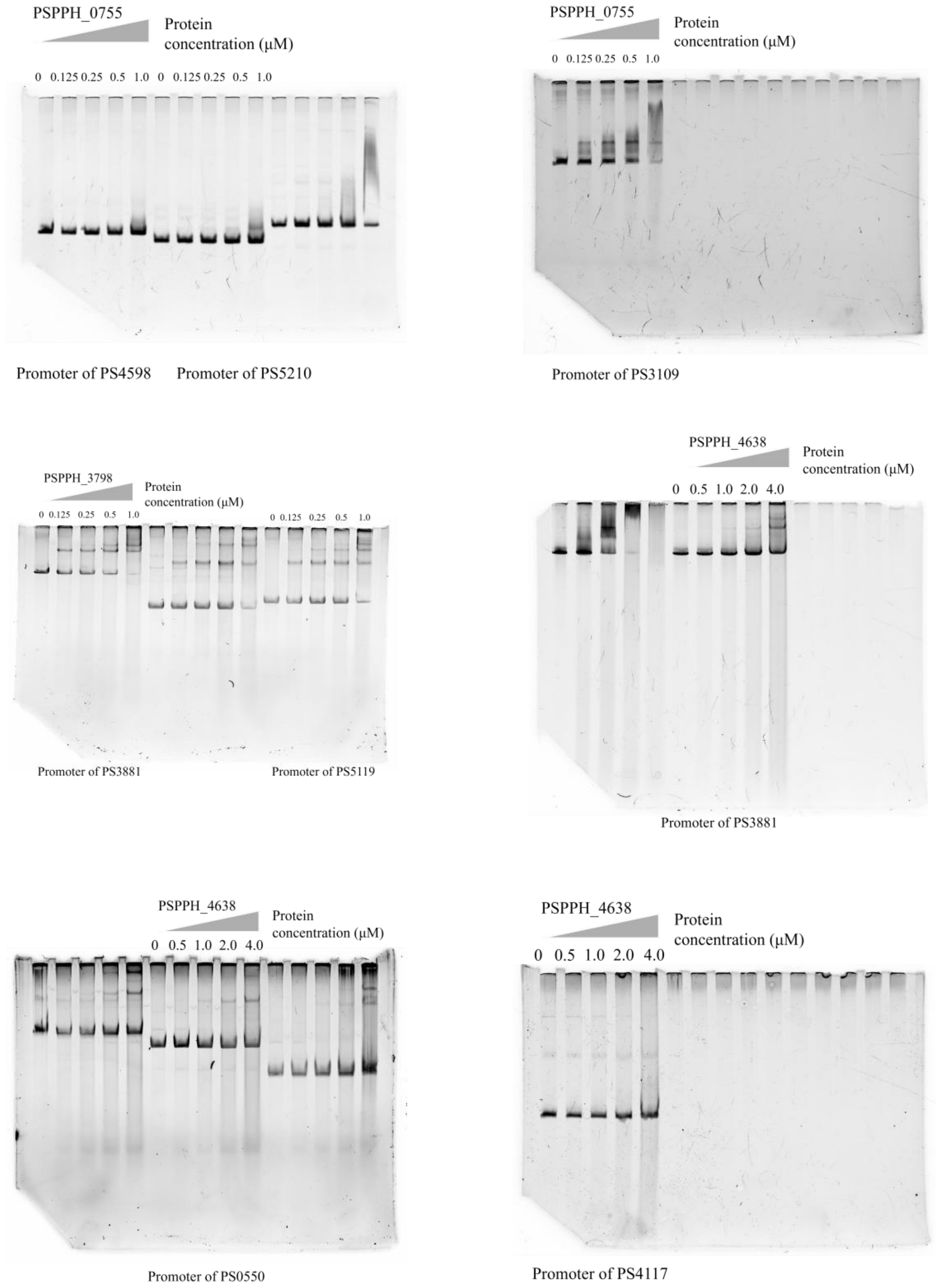

Figure 4-figure supplement 2-source data 1. Original gels corresponding to Figure 4-figure supplement 2-b-d. Protein concentration were labelled for EMSA.
